# Supplementary material for: Novel Driver Strength Index highlights important cancer genes in TCGA PanCanAtlas patients
Source: PeerJ. 2022 Aug 11;10:e13860. doi: 10.7717/peerj.13860 (PMC9375969; doi:10.7717/peerj.13860)
Supplement: Supplemental Information 2 [file peerj-10-13860-s002.zip › COHORTS/genes plots/age/2021_8_16_14_9_distribution_cohorts_females_70-74.pdf]

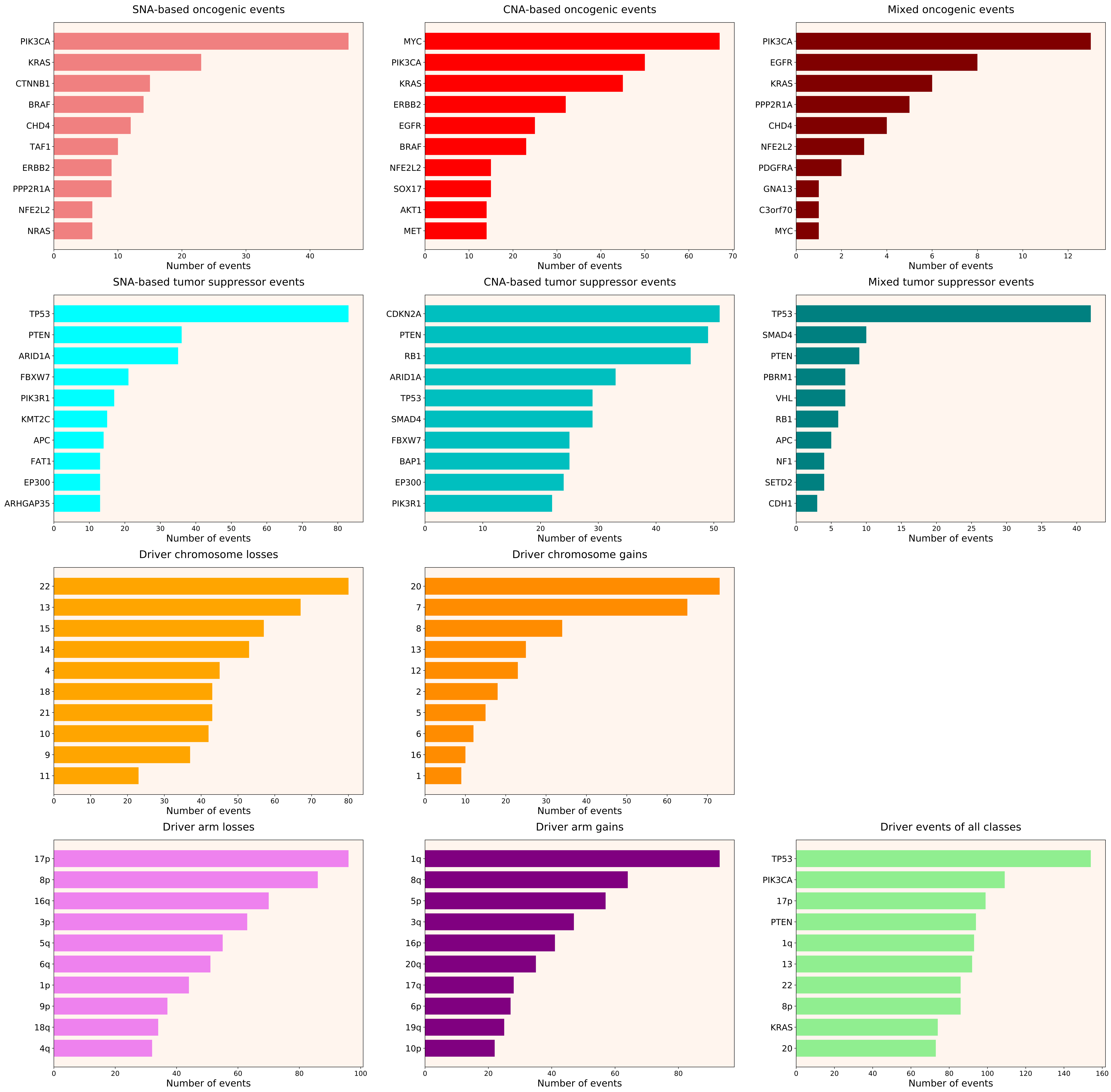
SNA-based oncogenic events
PIK3CA
KRAS
CTNNB1
BRAF
CHD4
TAF1
ERBB2
PPP2R1A
NFE2L2
NRAS
0 10 20 30 40
Number of events
CNA-based oncogenic events
MYC
PIK3CA
KRAS
ERBB2
EGFR
BRAF
NFE2L2
SOX17
AKT1
MET
0 10 20 30 40 50 60 70
Number of events
Mixed oncogenic events
PIK3CA
EGFR
KRAS
PPP2R1A
CHD4
NFE2L2
PDGFRA
GNA13
C3orf70
MYC
0 2 4 6 8 10 12
Number of events
SNA-based tumor suppressor events
TP53
PTEN
ARID1A
FBXW7
PIK3R1
KMT2C
APC
FAT1
EP300
ARHGAP35
0 10 20 30 40 50 60 70 80
Number of events
CNA-based tumor suppressor events
CDKN2A
PTEN
RB1
ARID1A
TP53
SMAD4
FBXW7
BAP1
EP300
PIK3R1
0 10 20 30 40 50
Number of events
Mixed tumor suppressor events
TP53
SMAD4
PTEN
PBRM1
VHL
RB1
APC
NF1
SETD2
CDH1
0 5 10 15 20 25 30 35 40
Number of events
Driver chromosome losses
22
13
15
14
4
18
21
10
9
11
0 10 20 30 40 50 60 70 80
Number of events
Driver chromosome gains
20
7
8
13
12
2
5
6
16
1
0 10 20 30 40 50 60 70
Number of events
Driver arm losses
17p
8p
16q
3p
5q
6q
1p
9p
18q
4q
0 20 40 60 80 100
Number of events
Driver arm gains
1q
8q
5p
3q
16p
20q
17q
6p
19q
10p
0 20 40 60 80
Number of events
Driver events of all classes
TP53
PIK3CA
17p
PTEN
1q
13
22
8p
KRAS
20
0 20 40 60 80 100 120 140 160
Number of events
